# Evaluating the impact of prediction models: lessons learned, challenges, and recommendations

**DOI:** 10.1186/s41512-018-0033-6

**Published:** 2018-06-12

**Authors:** Teus H. Kappen, Wilton A. van Klei, Leo van Wolfswinkel, Cor J. Kalkman, Yvonne Vergouwe, Karel G. M. Moons

**Affiliations:** 1 Division of Anesthesiology, Intensive Care and Emergency Medicine, University Medical Center Utrecht, Utrecht University, P.O. Box 85500, Mail stop F.06.149, 3508 GA Utrecht, The Netherlands; 2 Department of Public Health, Erasmus Medical Center, Rotterdam, The Netherlands; 3 Julius Center for Health Sciences and Primary Care, University Medical Center Utrecht, Utrecht University, Utrecht, The Netherlands; 4 Cochrane Netherlands, University Medical Center Utrecht, Utrecht University, Utrecht, The Netherlands

**Keywords:** Prediction models, Impact studies, Implementation, Diagnosis, Prognosis, Study design

## Abstract

An important aim of clinical prediction models is to positively impact clinical decision making and subsequent patient outcomes. The impact on clinical decision making and patient outcome can be quantified in prospective comparative—ideally cluster-randomized—studies, known as ‘impact studies’. However, such impact studies often require a lot of time and resources, especially when they are (cluster-)randomized studies. Before envisioning such large-scale randomized impact study, it is important to ensure a reasonable chance that the use of the prediction model by the targeted healthcare professionals and patients will indeed have a positive effect on both decision making and subsequent outcomes. We recently performed two differently designed, prospective impact studies on a clinical prediction model to be used in surgical patients. Both studies taught us new valuable lessons on several aspects of prediction model impact studies, and which considerations may guide researchers in their decision to conduct a prospective comparative impact study. We provide considerations on how to prepare a prediction model for implementation in practice, how to present the model predictions, and how to choose the proper design for a prediction model impact study.

**Electronic supplementary material:**

The online version of this article (10.1186/s41512-018-0033-6) contains supplementary material, which is available to authorized users.

## Background

Prediction models—both diagnostic and prognostic—are abundant in the medical literature [1–3]. A model that demonstrates adequate discrimination, calibration, and classification may be expected to have a good predictive performance in clinical practice. Nonetheless, this does not guarantee that actual use of the model in clinical practice will enhance medical decision making let alone improve health outcomes of the targeted individuals [4–6]. A model’s impact on decision making and subsequent health outcomes may be quantified in comparative—ideally cluster-randomized—prediction model impact studies [7–10]. In the index arm, healthcare workers use the prediction model in their practice, whereas in the control arm the healthcare workers do not use this model (i.e. are not exposed to its predicted probabilities). The actions of the index group that are guided by the model’s predicted probabilities are then compared to the actions of the control group that provides care-as-usual. The impact on subsequent health outcomes can also be compared between the two groups.

A cluster-randomized prediction model impact study may cost substantial effort and money [8–10]. The impact on health outcomes has only formally been studied for a very small proportion of the available prediction models [10–13]. There currently are too many prediction models to study the impact of each of them in large-scale cluster-randomized trials. Consequently, the medical community is stuck with large numbers of developed prediction models. How can we decide whether a model should be subjected to a randomized impact study, and if so, how to design such a study?

**Fig. 1:**
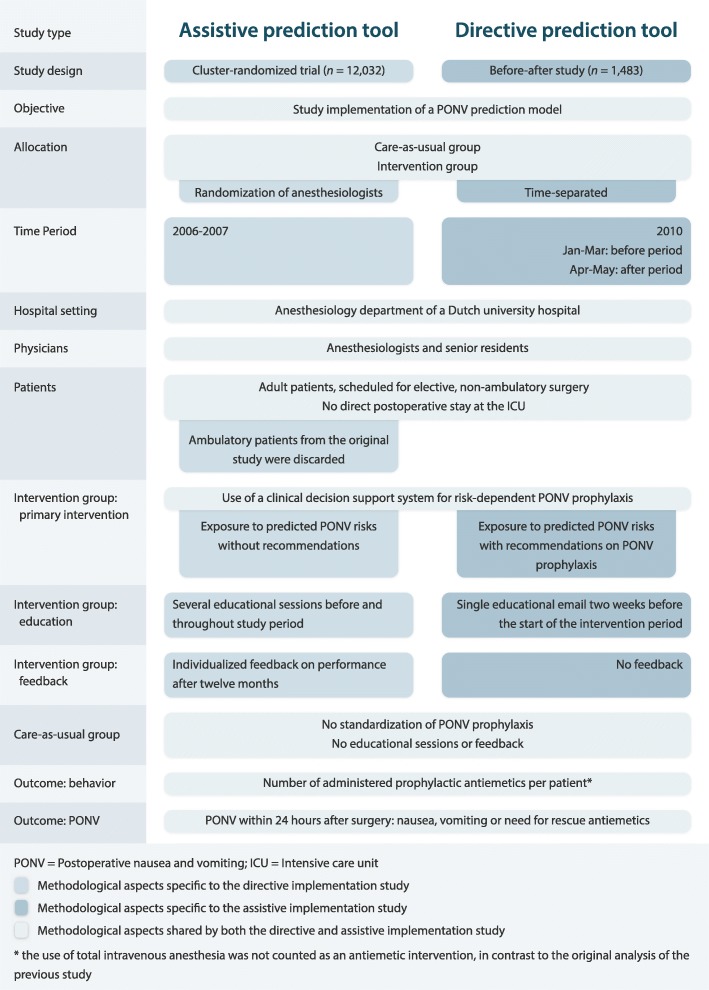
Methodological similarities and differences between our cluster-randomized trial with an assistive prediction tool (first study) and the before-after study with a directive prediction tool (second study)

We recently performed two differently designed, prospective comparative impact studies on a single prediction model. The model predicts the risk of postoperative nausea and vomiting (PONV), which surgical patients consider to be a very unpleasant side effect of anesthesia [14, 15]. The model is supposed to aid anesthesiologists in their decisions on pre-emptive PONV management (see Fig. 1 and Additional file 1) [16, 17]. The model was previously developed and externally validated (Table 1, left column) [18, 19]. Our two comparative impact studies had very different results and inferences, which in retrospect could have been expected and thus prevented. From both studies, we learned several valuable lessons from the challenges we faced with the different aspects of a prediction model impact study: whether the prediction model is ready for implementation (Table 2); how to present the model predictions (Table 3); and the design and analysis of the impact study (Table 4). The aim of this manuscript is to share these lessons. We regard them to be important considerations to guide researchers in their decision whether to conduct a new prediction model impact study, and if so, how to optimally design such a study.

**Table 1:** Predictors and regression coefficients of the original and updated models

Original model		Updated model	
Predictor	Regression coefficients		Predictor
Age (years)	− 0.022	− 0.017	Age (years)
Female gender	0.46	0.36	Female gender
Current smoking	− 0.63	− 0.50	Current smoking
History of PONV or motion sickness	0.76	0.60	History of PONV or motion sickness
Lower abdominal or middle ear surgery	0.61	0.48	Abdominal or middle ear surgery^a^
Isoflurane and/or nitrous oxide anesthesia^b^	0.72	0.35	Inhalational anesthesia^b^
	–	− 1.16	Outpatient surgery^c^
Intercept	0.15	0.12	Intercept
	Model performance characteristics^d^		
Model discrimination as C-statistic (95% CI)	0.62 (0.60–0.64)	0.68 (0.66–0.70)	Model discrimination as C-statistic (95% CI)
Calibration slope (95% CI)	0.57 (0.48–0.66)	1.00 (0.89–1.10)	Calibration slope (95% CI)

*PONV* postoperative nausea and vomiting, *CI* confidence interval

^a^In the updated model, the predictor included lower abdominal, upper abdominal, and laparoscopic surgery in addition to middle ear surgery

^b^As compared to intravenous anesthesia using propofol

^c^Predictor not included in the original model, but added in the update of the model

^d^Model performance was validated in a subset of patients (between March 2006 and February 2007) treated by anesthesiologists of the care-as-usual group of the cluster-randomized trial [19]

**Table 2:** Is the prediction model ready for implementation?

1. Assess the current state of scientific evidence	
A prediction model should at least have been validated once to assess its predictive performance in new patients or in a new setting. Subsequent diagnostic and therapeutic steps should also have a valid scientific base.	
2. Verify the predictive performance of the prediction model in the new setting	
Local practice, medical care, and patient population may not be similar to the setting in which the prediction model was derived. Consider possible differences between the two settings.	
3. Tailor the prediction model to optimize the predictive performance in the new setting	
An insufficient predictive performance in the new setting requires a model update. Even simple adjustments may overcome poor performance in the new setting.	
4. Develop a real-time strategy to handle missing predictor values when using the model	
Multivariable imputation is preferred over simply omitting predictors. Other predictors of the model, additional patient information, and information about the local clinical process may be used to estimate missing predictor values.

**Table 3:** How to present the model predictions?

Facilitators: features that increase the ease of use of a prediction model	
F.1 Add a decision recommendation to the predicted probabilities	
Directive prediction tools may be easier for physicians to use in their decision making than assistive prediction tools that provide only predicted probabilities without decision recommendations.	
F.2 Automatic calculation and presentation of the model’s probability within the physician’s workflow	
Minimizing manual predictor value entry and integrating the estimation of the model’s probability in the electronic patient record will facilitate the ease of use of a prediction model for care providers.	
F.3 Provide the reasoning or research evidence behind the predicted probability	
Enhances face value, acceptation and belief in the model, and thus the willingness to use the model’s probabilities to guide decision making.	
Barriers that may decrease the ease of use of a prediction model	
B.1 A predicted probability may be difficult to use in decision making, especially without corresponding recommendations	
Weighing the numerical probabilities with other available information will require more cognitive effort from physicians when the probabilities are presented without a corresponding recommendation on subsequent treatment or additional diagnostic testing.	
B.2 When the targeted physicians use an intuitive rather than analytical process of decision making	
When an existing decision-making process is mostly intuitive, it may require more cognitive effort to use probabilistic knowledge in decision making.	
B.3 When the predicted outcome is not a main concern for the physicians	
Physicians will not prioritize their time and efforts to use a prediction model in their decisions, when they consider other problems or outcomes to be more important.	
B.4 A prediction model does not weigh the benefits and risks of treatment or additional diagnostics regarding the patient’s (co)morbidity	
When a physician has more sources of information about the benefits and risks of subsequent treatment decisions, she/he will still have to weigh the model’s predicted probability from the model against this information, which is often perceived as cumbersome.

**Table 4:** Design of the impact study

1. Consider a decision analytic study	
If not previously performed, a decision analytic study may link the available evidence to estimate the theoretical impact on decision making and/or patient outcome.	
2. Consider studying the effects on both physician behavior and patient outcome	
Changes in process or behavior may not be sufficient to improve patient outcome. Studying the effects on patient outcome typically requires more time and money.	
3. Consider additional data collection to improve the understanding of the impact study results	
The impact does not only depend on the prediction model, but also on physician decision making and the effectiveness of subsequent treatment. Without additional data that is collected during the impact study, the effects of the individual components may difficult to disentangle.	
4. Compare the use of a prediction model to care-as-usual	
Physicians are not naive in patient selection and making interventional decisions. The impact of a prediction model (assistive or directive) is its value over and above current clinical decision making.	
5. Cluster-randomized trial as the optimal design	
Randomization of practices or practitioners aims to prevent learning effects and contamination between study groups. Nonetheless, time and costs to perform a cluster-randomized study should be weighed against its expected informational value.	
6. Consider using each study group as its own control	
The balance between study groups may be improved when using a stepped wedge design and including pre-trial observations.	
7. The impact of the prediction model will depend on the predicted probability^a^	
Predicted probability should be considered an effect modifier in the statistical analysis, which requires, e.g. stratification or use of its interaction term with ‘study group’ in regression analyses.	
8. All predictors should be available for care-as-usual patients^a^	
A probability-dependent analysis of the results requires that the predicted probabilities can afterwards also be estimated for the care-as-usual patients (control group). Accordingly, all predictors must be available for care-as-usual patients, even the costly or invasive predictor variables.	

^a^Additional item, not further explained in the manuscript

### Is the prediction model ready for implementation?

#### Does the current state of evidence warrant implementation?

Before a prediction model is implemented in clinical practice—within an impact study or not—it is imperative to ensure that the model is indeed ready for clinical use [6]. An adequate *development* of the model does not suffice. The model’s predictive performance should at least have been verified once in other individuals than from which it was developed in a so-called (external) *validation* study—ideally performed by other researchers (Table 2, item 1) [9, 11, 20]. When the model aims to guide medical decisions on subsequent interventions, the expected effects of these interventions should also have a solid scientific base.

In our example, the PONV prediction model had been developed and externally validated prior to its implementation studies. There was also sufficient scientific evidence on the health effects of the therapeutic interventions to prevent PONV, established by several randomized trials and meta-analyses on these interventions [21–24].

#### Model performance in the new setting

The new setting in which the model is implemented may be different from the setting in which the prediction model was derived or validated [5, 6, 25]. The local practices may be different in terms of both medical care and patient populations. If these differences are large, the prediction model may yield inaccurate risk predictions, lead to improper decisions and thus compromise patient outcomes in the new setting (Table 2, item 2). We would recommend that healthcare providers and researchers discuss possible differences between the settings of the preceding studies and the new setting of the impact study, before the model is implemented. When individual patient data from this new setting are available, the predictive performance of the model can and should first be validated in the new setting.

In our example, a discussion on the possible differences between settings proved fruitful. In a previous study, the model had validated adequately in the new impact setting [18]. However, we knew that two of the predictors of the original prediction model had changed over time and that our population would include both outpatients and inpatients, whereas the model was originally developed on inpatients only (Table 1) [19]. We therefore performed an update of the model to tailor it to the new setting.

#### Tailoring the prediction model to the new setting

When observed or expected differences between the new and preceding settings are large, the prediction model should be tailored to the new setting to overcome such differences, i.e. to optimize the predictive performance in the new setting and minimize the number of inaccurate predictions. This can be achieved by—for example—recalibration of the model (Table 2, item 3) [19, 26]. It often suffices to simply adjust the baseline risk or hazard of the model to the baseline risk or hazard found in the new setting [26, 27]. However, tailoring requires individual patient data—both on predictors and outcome—from the new setting to be available. Recent scientific efforts are exploring how differences between settings actually affect a model’s performance when validated or used in another setting [25, 28, 29]. This has not yet resulted in a clear set of validation guidelines on when model performance will be adequate in the new setting or when further tailoring of the model is necessary. Until clear guidelines exist regarding the number of external validations that are needed before use in daily practice, we recommend to first quickly evaluate the need for customization of the model to the new setting in which the model will be implemented, even when a model has been thoroughly validated in other settings.

In our example, we used local individual patient data to adjust the original baseline risk and weights of the predictors, and we added a new predictor to the original model to overcome the expected differences (Table 1, left versus right column). This improved both calibration and discrimination of the model in our local setting. At the time, we considered the discriminative ability to be adequate with a C-statistic of 0.68, because other models had proven to have similar performance [30]. Nonetheless, even with a moderate discriminative ability, a properly calibrated model would enable physicians to identify specific risk groups of patients and increase the number of prophylactic interventions according to the predicted risks. For details on this update, we refer to our preceding publication [19].

#### Handling missing predictor values when using the model

At the moment of probability calculation, one or more predictor values may be missing (unobserved) for an individual. A model may have been derived from a prospective cohort study, whereas it will be implemented in daily practice with possibly a lower quality of data collection. Healthcare workers do not always capture the same full set of signs, symptoms and lab values, or a device that is necessary to measure one of the predictor values is unavailable. In our example, predictor values for our model were only a small part of all the information that was gathered from patients during preoperative outpatient evaluation. The PONV risk was automatically calculated by the electronic patient record during anesthesia. As the patient was anesthetized, the physician was not able to complete missing predictor values. In the event of missing data, the model’s probability cannot be estimated at the time of decision making. A real-time strategy to impute this missing value is preferred over simply omitting the predictor from the model and over imputing an overall mean value of that predictor (Table 2, item 4) [19, 31]. Such real-time imputation requires a dataset that allows the development of the necessary imputation models. Auxiliary information may be used to improve the imputation models [19].

In our example, we developed imputation models to predict missing (unobserved) values of the predictors ‘high-risk surgery’, ‘smoking status’, and ‘history of PONV or motion sickness’ using all other available patient information. The predictor ‘high-risk surgery’ was commonly missing because the surgical procedure was only available as free text. We therefore used the surgical service of the procedure (e.g. Vascular or Gynecology) to impute missing values for the type of surgery being an abdominal or middle ear procedure, which are procedures that increase the risk of PONV (see Table 1). For details on how this imputation model was developed and used in real practice, we refer to our preceding publication [19].

### How to present the model predictions?

#### Assistive versus directive format

When planning a prediction model impact study or implementing a model in daily practice, one needs to decide how model predictions will be presented to its potential users. In an assistive approach, predictions are simply presented as numerical probabilities without corresponding decision recommendations. In a more directive approach, predictions are presented as decision recommendations which may or may not include the numerical probabilities [7, 9]. An assistive presentation format leaves more room to combine predictions with clinical judgment [32–34]. Current literature suggests that a directive format has greater impact on decision making and thus health outcomes than an assistive format [7, 9, 32–34].

However, there is hardly any empirical evidence for this suggestion. There are yet no studies that provide a more direct comparison between an assistive versus a directive format for a specific prediction model in a single setting—i.e. a single population of physicians and patients. Our two prospective, comparative model impact studies have given us the opportunity to provide this evidence, albeit from two subsequent studies in the same setting.

**Fig. 2:**
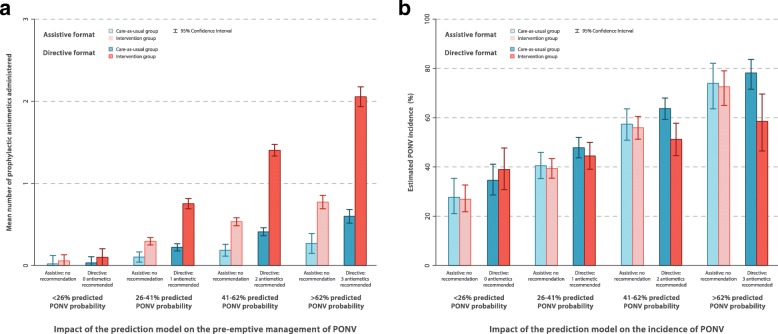
Graphical representation of the mixed effects regression analysis on the pooled dataset of the two impact studies. In both studies, a prediction model for postoperative nausea and vomiting (PONV) was implemented (red colors) and compared to care-as-usual (blue colors). In one study, the probabilities of the model were simply presented to physicians who implemented without a recommendation (i.e. an assistive format; less saturated colors). The other study also included an actionable recommendation (i.e. a directive format; more saturated colors). Both studies compared the impact on the physicians’ administration of antiemetic prophylaxis (**a**) and on the incidence of PONV (**b**). The bars and their 95% confidence intervals (CI) represent the fixed effects of the mixed effects regression analyses. The mixed effects models included fixed effects for the following variables: study, allocation group, predicted probability of PONV, and all interaction term between these variables. Because of the similarity of the results, the bars were calculated from the unadjusted analysis after multiple imputations. The 95% CIs were calculated from the covariance matrix for the variable study, allocation group, predicted probability, and their interaction terms. Further methodologic information and the numerical results of the regression models are available in Additional file 1

Our first study was a cluster-randomized trial in which we randomized 79 physicians of the Anesthesiology Department at the University Medical Center Utrecht, The Netherlands, who together treated over 12,000 surgical patients within 2 years (see Fig. 1, left column) [16]. For the intervention group physicians, predicted PONV probabilities for each of their patients were presented on-screen during the entire procedure, but without corresponding recommendations on the number of prophylactic antiemetics to administer. The control group physicians performed care-as-usual (no ‘exposure’ to predicted PONV probabilities). Significantly more prophylactic antiemetics were administered by the intervention group physicians (Fig. 2a). Unexpectedly, this increase was not accompanied by a decreased PONV incidence (Fig. 2b). The prediction tool indeed changed physician behavior, but thus did not improve subsequent patient outcomes.

Secondly, prompted by this result, we conducted a subsequent prospective model impact study, employing a non-randomized before-after design. We implemented the prediction model for a second time in exactly the same setting, comparing clinical practice and patient outcome during the periods before and after implementation of the intervention. This time we added a treatment recommendation to the predicted probability. The recommendations were largely based on existing international guidelines on PONV prophylaxis, which already recommended risk-based PONV prophylaxis using a prediction model (Fig. 1) [17, 35]. The impact of the directive prediction tool could then directly be compared to the impact of the assistive tool of the preceding cluster-randomized trial. In contrast to the assistive prediction tool, we found that the directive prediction tool not only substantially improved decision making but also improved patient outcomes (Fig. 2). As this was a before-after study, there is always the possibility that unobserved time effects may be the underlying cause of the observed differences. For example, of the 42 attending physicians who treated patients during the before-after study, 34 were part of the randomization of the cluster-randomized trial. As these 34 physicians received the results of the cluster-randomized trial, it is possible that this may have increased antiemetic prescription during the before-after study. Nonetheless, a large, risk-dependent change in decision making with a corresponding risk-dependent change in patient outcome makes it quite plausible that the observed difference between the before and after periods is caused by the intervention [36]. From a study design perspective, a prediction model impact study should be regarded as a program evaluation, in which the implementation of a complex intervention is studied [37].

To our knowledge, this is the first comparison between a directive and an assistive format of the same prediction model within a single setting, demonstrating that a directive format not only has a greater impact on clinical practice but also on patient outcomes (Table 3, F.1).

#### Addressing the physician’s perspective

To understand how physicians use predicted probabilities in their decision making, we also performed face-to-face interviews and a structured online survey among the participating physicians in the cluster-randomized trial.

During the interviews, possible facilitators and barriers for the use of risk-based PONV prophylaxis were explored. The survey was used to quantify how often these facilitators and barriers were present and to identify possible differences between ‘exposed (index)’ and ‘non-exposed (control)’ physicians. We found that using a prediction tool requires substantial cognitive effort from physicians (Table 3, B.1), notably when such decision making is usually an intuitive rather than analytical process (Table 3, B.2). It seemed not trivial to assist predicted probabilities with specific therapeutic recommendations, especially in a high-workload environment such as surgical, emergency or critical care settings (Table 3, B.3). Physicians reported that adding decision recommendations to predicted probability categories may be a way to decrease the required cognitive effort and increase the ease of use of the prediction tool, as supported by our results (Table 3, F.1) [38].

This does not mean that probabilities alone cannot be useful. In clinical settings where decision making is already probabilistically oriented, introducing a new model might not increase the required cognitive effort to carefully interpret probabilities and make subsequent decisions. Consequently, when designing a model impact study one should understand the new setting beforehand—e.g. by including clinical champions or conducting a small survey among potential users. The presentation of the prediction model probabilities with or without recommendations should be tailored to the needs of its potential users, in our case the anesthesiologists [39]. Automatic provision of the predicted probabilities and smooth integration within the current physician’s workflows are key factors to improve understanding, compliance, usability, and thus the effects of the model use on decision making and subsequent health outcomes (Table 3, F.2) [33, 34].

Physicians also use additional information in their decisions, rather than solely relying on the information presented by the prediction tool (Table 3, B.4). In our example, such information included other risk factors for PONV that are not included as predictors in the model—e.g. expected opioid use—or patient comorbidities that may increase the risks of side effects from specific antiemetic drugs—i.e. the risk of arrhythmias or hyperglycemia. We learned that it is essential that the potential users should be well informed about the underlying assumptions of the model—e.g. which predictors are in the model and why. This will enhance their understanding of predicted probabilities and their adherence to corresponding management recommendations (Table 3, F.3) [33, 34, 40]. When physicians are more aware of the contents of the model, they may also better understand how the possible interventions may affect patients with specific outcome risk profiles. When the prediction model largely remains a black box to the physicians, they may not acknowledge the mechanisms underlying the predicted probabilities and select the wrong intervention or no intervention at all. In such cases, a prediction tool may do more harm than good. When aware of such phenomena, one may include information on underlying assumptions of the model in the presentation and format of the prediction model [4, 37]. Further study is needed on what the best way is to inform physicians in the underlying assumptions and mechanisms of a prediction model that is being implemented.

### Design of the impact study

Cluster-randomized prediction model impact studies can be very costly and time-consuming. Before initiating one, it is important to contemplate whether such study will be worthwhile. We discuss three major considerations when designing such study: decision analytics, the choice of the outcome variable(s), and the study design.

#### Consider to first do a decision analytic study

Before proceeding to conduct a model impact study of a validated model—let alone the implementation in daily practice—the possible impact on decision making may be first estimated by so-called decision analytic studies (Table 4, item 1) [41–44]. Decision analytic techniques can estimate how the model’s predicted probabilities will affect the decisions, what the expected effects of the subsequent treatments are on patient outcomes, and which predicted probability thresholds may best be used to start or withhold certain intervention possibilities. The inferences on the potential impact of the prediction model are thus based on the model’s predictive accuracy in combination with the effectiveness of subsequent interventions. It further requires additional assumptions on how predictive information is used by patients and clinicians in their decision making: how patients and clinicians weigh the importance of false positives and false negatives, and how they will translate those weighted risks into treatment decisions. It is also possible to include the rate of side effects of the potential interventions in decision analytical models. Finally, the effects of possible variability in predictive accuracy, intervention effectiveness, and decision making can also be modeled in decision analytic techniques.

Decision analytic studies thus indicate whether the use of a prediction model is indeed likely to impact health outcomes and thus may be studied in a (cluster-)randomized impact study. Moreover, such studies may indicate which subgroups may respond best, which probabilities with corresponding treatment combinations may be chosen, and which model and treatment adherences need to be reached. Models that are likely to have no impact should not proceed to prospective comparative impact studies. Accordingly, preceding decision analytic studies help in separating the chaff from the wheat [45].

#### Studying patient outcome and not only effects on decision making

The chosen outcome affects the necessary resources of a randomized model impact study in two ways: the resources to collect the data per study participant and the required number of study participants. A change in process variables—e.g. change in administered treatment—is typically easier to study than a change in patient outcome measured later in time. Moreover, expected changes in process variables are usually larger than expected changes in outcomes measured later in time. The required sample sizes will thus be smaller for process variables. The further in time the expected effects of the therapeutic interventions are, the more cumbersome an impact study will be.

Making use of routinely collected data is also a good way to reduce costs. Process variables are often part of routinely collected data in contrast to many patient outcomes, although national registries or institutional audits may also provide the necessary patient outcomes and reduce costs. One should always be aware of possible data quality issues in routinely collected data as the data is collected for a different purpose.

Even though PONV is a reasonably efficient patient outcome (high incidence, large effect by administered treatments, and occurring shortly after the prediction model use), it still required a larger sample size than would we have chosen physicians’ decisions as the primary outcome.

Nonetheless, one should realize that introducing a prediction model with subsequent management actions is an introduction of a complex intervention [4, 46]. The entire intervention consists of multiple components that interact: the accuracy of the model predictions, physician and patient understanding of probabilities, expected therapeutic effects of administered treatments, and adherence to predicted probabilities and administered treatments. Consequently, the effects of the model use on downstream patient outcomes are not simply the sum of the consecutive components [37]. Changes in decision making processes or behavior may not always be sufficient to improve outcome. In our example, there was no decrease in PONV incidence, despite an increased administration of prophylactic antiemetics in the cluster-randomized trial of the assistive format (Fig. 2a, b). From a resource perspective, it may thus prove worthwhile to only measure the impact of the prediction model on behavior and perhaps estimate the subsequent effects on patient outcome through a decision analytical or linked-evidence model. However, the downstream effects on patient outcome of using a prediction model in clinical practice are not always predictable. This unpredictability increases when the outcomes are rare or occur later in time. Nonetheless, studying process outcomes and modeling the effects on patient outcome may be a valuable step to decide on the probability thresholds to start or withhold an intervention, even when the unpredictability is large. The challenge is to determine when studying patient outcome in an impact study is indeed necessary (Table 4, item 2).

Finally, the observed discrepancy in our results indicates that either the predictive performance of the model was insufficient, the impact on physician decision making was still too small (e.g. too few prophylactic drugs were administered despite high predicted probabilities), the antiemetic drugs were not as effective as thought, or a combination of these causes. As we studied the model’s predicted probability, physician decision making and the actual treatment effects as a ‘package deal’ intervention, the individual contribution of each in explaining the discrepant results could not be disentangled. When we tried to quantify the contribution of a specific component, we always had to make one or more assumptions on how the other components affected individual patients. For example, we had to make assumptions on the prediction errors for individual patients to estimate the antiemetic effectiveness, or assumptions on individual treatment effects of (specific) antiemetic drugs were required to estimate the accuracy of the prediction model. In our example, the only way to improve our understanding of the results of our cluster-randomized trial was through either additional data collection (interviews and surveys) or through further study (the directive impact study). When designing the impact study, we would recommend considering what possible data could be collected to improve the understanding of the study’s results, especially when the results are not unequivocally positive (Table 4, item 3).

#### Choosing a study design

The most important feature of a prediction model impact study is that, regardless of the presentation format, the exposure to the prediction model (index group) is compared to a similar group of physicians that is not exposed to the model and its predictions (control or care-as-usual group) (Table 4, item 4). Depending on the aim of the impact study, the prediction model may also be compared to other predictive aids or interventions, rather than being compared to care-as-usual. For such a comparison, it is very important that the study groups are comparable for all aspects other than the intervention. A randomized study design is the most effective way of achieving such balance, but such designs can thus greatly increase the required resources of a study. There are various other considerations when designing a (randomized) impact study [8, 10].

One first needs to consider whether the intervention will have an individual effect on patients or whether it induces a more group-like effect. A new drug under study will only affect the individual to which it is administered, but a prediction model often aims to affect the clinical routine of a physician, which may vary per physician. In prediction model impact studies, this leads to clustering of the effect per physician or per practice (hospital) when the model use is compared across providers or practices [8, 10].

Physicians may also become better at using the prediction model over time. In a non-clustered, randomized trial, where patients are randomized to either the intervention or care-as-usual group, physicians would encounter patients from both groups. After repeated exposure to the predictions in a variety of index group patients, physicians may become better at estimating the probability in subsequent similar patients, even when these patients are part of the control group [8, 9]. This likely dilutes the effectiveness and thus impact of the model use [47]. The effects of a learning curve may be minimized, though not completely prevented, by randomization at a cluster level, e.g. physicians or hospitals (Table 4, item 5) [48]. Because healthcare providers very often work in teams, contamination is much more likely to occur when healthcare providers are randomized than when, e.g. hospitals are randomized. In a cluster-randomized study, physicians of the intervention group may still experience a learning curve, but this does not necessarily lead to a dilution of the contrast between the two groups, but rather in a change in (improved) effectiveness over time.

A drawback of randomization at the cluster level is that one often requires a larger sample size. More efficient alternatives are non-randomized before-after studies or interrupted time-series studies, which compare a period without the model to a period with the model, as in our example [8, 10]. Similarly, practices where a model is being used may be compared to practices where it is not being used (parallel groups design). The challenge in such designs is to adjust for baseline differences between the two groups [8, 9]. Also, one may first study how a prediction model use changes treatment decisions as compared to a control group, following a cross-sectional design—even in a randomized fashion. If the decision making is not changed in the index group compared to the control group, it seems less intuitive to start a longitudinal impact study on patient outcomes [8, 9]. Although all these alternatives are more prone to bias, a negative result—i.e. no observed differences across study groups—may indicate that a cluster-randomized impact study focused on patient outcomes is not (yet) warranted.

In a cluster-randomized trial design, it can also be difficult to achieve balance between the intervention and control group. In our example, 79 physicians were randomized. As there was a large variation in the number and type of surgical patients each physician treated during the study, seemingly small baseline imbalances at the physician level caused substantial imbalances at the patient level. The care-as-usual group treated 53% of the patients of which 27% were outpatients, as compared to 47 and 38%, respectively, for the intervention group. The balance of a cluster-randomized trial can be improved by including crossovers in the study design, such as stepped wedge designs and cluster-randomized before-after studies, where each cluster has a time period with and without the intervention (Table 4, item 6) [49–52]. Each cluster can then also serve as its own control, enhancing the balance between study groups.

Would we be able to redo our cluster-randomized trial, we might consider doing pre-trial observations of the potential users and their decision making behavior [36, 53, 54]. In our example, physicians of the care-as-usual group also provided probability-dependent PONV prophylaxis to their patients without explicitly using a prediction model (Fig. 2). Although this may simply represent the clinical expertise of the physicians, once the study is completed, one cannot distinguish this from any Hawthorne effects or contamination between study groups. Observing the (care-as-usual) behavior of all physicians before the start of the trial has the advantage that one is able to quantify how physicians’ decision making changes within each study group (Table 4, item 5) [34, 53]. Such pre-trial observations would also have enabled us to verify whether physicians who are more inclined to treat PONV were indeed well balanced between the two study groups.

## Concluding remarks

Evaluating the impact of using a prediction model in a large-scale comparative study requires a phased approach. That approach should be tailored to each specific setting in which the model will be used or studied on its impact. The prediction model should be applicable to patients of the new setting in which it is implemented. The format of the prediction model—e.g. assistive or directive—should be carefully chosen and designed. Knowledge of the current behavior of the intended users, and their perspectives on risk-prediction models in general, is extremely helpful to determine how to best present the model, design the study and interpret its results.

Currently, the number of published prediction models is overwhelming [3, 10, 55]. It is simply impossible to study all these prediction models in large-scale cluster-randomized impact studies. We believe it is only cost-effective to perform a prospective comparative impact study when there is a reasonable chance to find a relevant positive effect on decision making and patient outcome. An adequate validation of the model in the new setting, and a positive decision analytical study indicate that the model is potentially effective. Hence, such tools may help to decide whether or not to proceed to a prospective comparative impact study. The results from these analyses may also be used to plan the subsequent impact study. The results may help to optimize the design of the impact study—e.g. to select appropriate probability thresholds for the intervention—and to improve the study’s analysis—e.g. which additional information needs to be documented.

## Additional file

Additional file 1: Supplemental digital content. (DOCX 56 kb)

## Abbreviations

CI: Confidence interval
PONV: Postoperative nausea and vomiting
