# Supplementary material for: Evaluating the impact of prediction models: lessons learned, challenges, and recommendations
Source: Diagn Progn Res. 2018 Jun 12;2:11. doi: 10.1186/s41512-018-0033-6 (PMC6460651; doi:10.1186/s41512-018-0033-6)
Supplement: Supplementary file 1 — Supplemental digital content. (DOCX 56 kb) [file 41512_2018_33_MOESM1_ESM.docx]

Methods and Results Supplement for “Evaluating the impact of prediction models in clinical practice: challenges and recommendations”

This supplement describes the comparison of two consecutive impact studies in which we implemented a specific prediction model for postoperative nausea and vomiting (PONV) in two different formats.[1, 2] The first format was an ‘assistive’ approach – i.e. presenting predicted risks to physicians without any therapeutic recommendations – and the second format was a ‘directive’ approach – i.e. presenting predicted risks accompanied with non-obligatory, therapeutic recommendations on prophylactic PONV management. This supplement describes how we contrasted the findings of the assistive approach to the findings of the directive approach. The methods and results for the individual studies are summarized in this supplement and a more elaborate description is available in the original manuscripts.

# Methods

#### Design and participants

The first impact study aimed to quantify the effect of the implementation of an assistive approach on both the administration of antiemetic prophylaxis and the incidence of PONV.[1] This study was a cluster-randomized trial, in which physicians (i.e. Anesthesiologists) were randomized to either the assistive prediction model or to care-as-usual. The study was conducted at the Anesthesiology Department of a Dutch university hospital (UMC Utrecht) in 2006-2007.

The second impact study aimed to quantify the effects of a directive PONV prediction model approach.[2] It was a prospective before-after cohort study, conducted at the same Anesthesiology Department in 2010. An overview of methodological differences between the two studies is presented in Figure 1 of the manuscript.

All adult patients undergoing general anesthesia for elective, non-ambulatory surgery who had visited the outpatient preanesthesia evaluation clinic were eligible for the present study. Both ambulatory and non-ambulatory patients were enrolled in the assistive study. To compare the effectiveness of the directive versus assistive prediction model approach, all ambulatory patients from the assistive study were excluded for the present analyses.

Exclusion criteria were pregnancy, postoperative admission to the intensive care unit, overnight ventilation at the postanesthesia care unit, and inability to communicate in Dutch or English. All eligible patients from the time of study initiation were automatically included using the anesthesia information management system.

#### The prediction model

The implemented prediction model was originally developed in a population of a different university hospital in the Netherlands and had already been externally validated.[3, 4] The model was subsequently updated and optimized for implementation at the UMC Utrecht, where both the present study and the previous randomized study took place.[5] The model consisted of seven predictor variables: age; gender; current smoking; type of surgery; inhalational anesthesia; ambulatory surgery; and history of motion sickness or PONV (for a full model description see Table 1 of the manuscript).

#### Intervention

###### Intervention group

The prediction model was implemented as a decision support tool in the anesthesia information management system (AnStat®), a custom-made system written by one of the authors (LvW). The model presented a patient’s predicted PONV risk as a percentage without any categorization. For the directive study, this risk was categorized in four risk groups and was accompanied with an advice on the number of prophylactic antiemetics to administer based on that individual’s risk. The anesthesia information management system automatically presented this risk and the recommendation to the responsible physician on the computer screen during the anesthetic case. In both studies, the physicians then decided if, which and how many prophylactic antiemetics would be administered in view of the patient’s individual risk or whether to follow the advice and administer prophylactic antiemetics accordingly.

The assistive study included several educational sessions before patient enrollment, at the start and throughout the study period. In addition, the physicians received individualized feedback by email after the first twelve months of the assistive study. For the directive study, the implementation of the decision support tool was announced to all physicians and anesthetic nurses in an email from the department head two weeks before the start of the intervention period, including an attached instruction on the use of the decision support tool. No other educational sessions or feedback reports were employed during the directive study.

###### Control group

The control group was defined as care-as-usual, achieved by randomization of physicians to care-as-usual in the assistive study or care-as-usual in period before the implementation of the intervention in the directive studied. Physicians of the control group were not exposed to any automated prognostic information by a prediction model. Prophylactic management of PONV was not standardized in any way, which was according to care-as-usual in our hospital.

#### Outcome and follow-up

The change in administration of risk-dependent PONV prophylaxis (physician behavior) was defined as the number of prophylactic antiemetics administered per patient and was recorded in the anesthesia information management system. In contrast to the assistive study, the use of total intravenous anesthesia was not counted as a prophylactic intervention regarding the primary outcome, as it was unlikely to change during the anesthetic case. However, as inhalational anesthesia was a predictor within the prediction model, the presented recommendation did depend on the type of anesthesia used. There was no active surveillance of adverse events during this study.

The incidence of PONV (patient outcome) was defined as the occurrence of nausea, vomiting or the need for rescue antiemetics within 24 hours after surgery. Nausea was rated on a three-point verbal rating scale (no / yes a bit / yes definitely). Vomiting was recorded as a binary outcome (no / yes). Research nurses and trained medical students collected data on the occurrence of postoperative nausea using a validated questionnaire.[3, 6] Data were collected at the post anesthesia care unit (30 minutes and 60 minutes after arrival, and when leaving the unit), and 24 hours after surgery on the ward. The outcome variable for PONV was coded as missing when any of the follow-up measurements had not been completed.

#### Statistical analysis

The data of the assistive study and of the directive study were merged to quantify the impact of a more directive implementation of the prediction model on clinical practice as compared to an assistive approach. Analysis was performed under the intention-to-treat principle. Mixed effects regression analyses were used for both outcomes: linear regression for the number of prophylactic antiemetics per patient and logistic regression for the incidence of PONV (glmer, lme4 library, R software). A random intercept was included in the models, as the studies were clustered by anesthesiologists. For both outcomes, the ‘study’, (assistive versus directive study), allocation group (intervention versus care-as-usual), predicted PONV risks, and the interaction between all three variables were included as independent variables in the model. The interaction terms were included to quantify to what extent the difference in treatment effect (between assistive and directive, and between intervention and care-as-usual) differed across predicted risks; e.g. an odds ratio below one would signify that a reduction in PONV due to the directive approach was greater in patients with higher risks.

For the analysis of physician behavior, we expected a linear association between the outcome (actual number of administered prophylactic antiemetics, ranging from 0 to 3) and intervention (recommended number of antiemetics per predicted PONV risk category). Therefore, not the continuous predicted risk variable but rather the advised number of antiemetics and its interaction with allocation group were used as independent variables in a linear regression model with the administered number of antiemetics as the dependent variable. For the PONV incidence analysis predicted PONV risks were included as a continuous variable.

As this is analysis is a non-randomized study, we had to adjust for potential differences between various groups. Although inclusion of the predicted risk variable and its interaction term with study groups would probably adjust for most of the confounding, we a priori hypothesized to adjust additionally for all variables from Table 1in the manuscript (PONV risk factors) and for ASA class and procedure duration.[7]

Before multivariable modeling, all continuous variables were tested for nonlinearity using restricted cubic splines, including predicted PONV risk.[8] Missing data were multiply imputed (n=10) using a regression approach in R (aregImpute, library Hmisc). Imputation of missing variables was based on predictors, outcome variables, and other perioperative data.[9–11] As PONV was coded missing when any of the follow-up measurements was incomplete, non-missing follow-up measurements of PONV were added to the imputation process to serve as auxiliary variables to impute missing values for PONV. Subsequently, the imputed values for PONV were included into the mixed effects regression model, instead of deleted. The anesthesiologists were added as an extra variable in the imputation model to take into account clustering in the data.

# Results

Baseline differences for both studies are displayed in Table 1. In contrast to the assistive study, the directive approach in the before-after study resulted in a larger increase in risk-dependent prophylaxis than the assistive approach in the earlier trial (Table 2, both panels). Following this increase in prophylaxis administration, there was a decrease of PONV in patients with a predicted PONV risk greater than 41% (2 or 3 antiemetics recommended, see Table 3). The results of the regression models of the comparison between studies – on which Figure 2 of the manuscript is based – can be found in Tables 4 and 5.

# Table 1 – Patient characteristics of the assistive study (left) and the directive study (right)

|  | | **Assistive Study** | | | | **Directive Study** | | |
| --- | --- | --- | --- | --- | --- | --- | --- | --- |
|  |  | | Care-as-Usual Group | Intervention Group |  | | Care-as-Usual Group | Intervention Group |
|  | N* | | (*n* = 4496) | (*n* = 3385) | N* | | (*n* = 1022) | (*n* = 461) |
| Age, mean (SD), years | 7881 | | 52 (17) | 52 (17) | 1483 | | 52 (18) | 54 (18) |
| Female gender, No. (%) | 7881 | | 2353 (52) | 1782 (53) | 1483 | | 496 (49) | 195 (42) |
| ASA class, No. (%) | 7691 | |  |  | 1480 | |  |  |
| 1 |  | | 1501 (34) | 1110 (34) |  | | 337 (33) | 121 (26) |
| 2 |  | | 2312 (53) | 1772 (54) |  | | 561 (55) | 274 (60) |
| 3 |  | | 558 (13) | 419 (13) |  | | 116 (11) | 62 (14) |
| 4 |  | | 14 (0) | 5 (0) |  | | 8 (1) | 1 (0) |
| Current smoking, No. (%) | 7533 | | 1272 (30) | 963 (30) | 1456 | | 288 (29) | 111 (25) |
| Surgery with a high PONV risk, No. (%) | 7162 | | 751 (19) | 484 (16) | 1106 | | 82 (12) | 53 (13) |
| History of PONV / Motion sickness, No. (%) | 5736 | | 1034 (32) | 806 (32) | 1398 | | 212 (22) | 66 (15) |
| Inhalational anesthesia, No. (%) | 7881 | | 1720 (38) | 1418 (42) | 1483 | | 472 (46) | 226 (49) |
| Predicted risk of PONV, mean (SD) | 7881 | | 0.42 (0.13) | 0.42 (0.13) | 1483 | | 0.40 (0.13) | 0.39 (0.12) |
| Predicted PONV risk in categories, No. (%) | 7881 | |  |  | 1483 | |  |  |
| <26% (0 antiemetics advised^†^) |  | | 449 (10) | 316 (9) |  | | 127 (12) | 61 (13) |
| 26-41% (1 antiemetic advised^†^) |  | | 1725 (38) | 1301 (38) |  | | 443 (43) | 218 (47) |
| 41-62% (2 antiemetics advised^†^) |  | | 1970 (44%) | 1503 (44) |  | | 384 (38) | 162 (35) |
| >62% (3 antiemetics advised^†^) |  | | 352 (8) | 265 (8) |  | | 68 (7) | 20 (4) |
| Operation duration, median (IQR), min^‡^ | 7881 | | 126 (82-192) | 87/126/(87-191) | 1483 | | 128 (85-188) | 167 (119) |
| * N represents the total number of non-missing observations for that characteristic; note that for the current analysis ambulatory patients have been removed from the assistive study  ^†^ Advice only presented to physicians of the intervention group in the directive study  ^‡^ Median with interquartile range is presented as the distribution for operation duration is skewed  PONV = postoperative nausea and vomiting; SD = standard deviation; IQR = interquartile range | | | | | | | | |

# Table 2 – Crude risk-dependent administration of antiemetic prophylaxis between allocation groups of both impact studies

|  | | **Assistive Study** | | | **Directive Study** | | |
| --- | --- | --- | --- | --- | --- | --- | --- |
|  |  | | Care-as-Usual Group^†^ | Intervention Group^†^ |  | Care-as-Usual Group^†^ | Intervention Group^†^ |
| Predicted PONV risk in categories | N* | | (*n* = 4496) | (*n* = 3385) | N* | (*n* = 1022) | (*n* = 461) |
| <26% (0 antiemetics advised^‡^) | 765 | | 0.11 (0.32) | 0.12 (0.35) | 188 | 0.079 (0.27) | 0.12 (0.32) |
| 26-41% (1 antiemetic advised^‡^) | 3026 | | 0.12 (0.34) | 0.27 (0.52) | 661 | 0.21 (0.48) | 0.74 (0.53) |
| 41-62% (2 antiemetics advised^‡^) | 3473 | | 0.22 (0.45) | 0.53 (0.64) | 546 | 0.42 (0.68) | 1.5 (0.85) |
| >62% (3 antiemetics advised^‡^) | 617 | | 0.30 (0.51) | 0.82 (0.69) | 88 | 0.68 (0.70) | 1.9 (1.1) |
| * N represents the total number of non-missing observations for that characteristic  ^†^ Data represent mean number of prophylactic antiemetics (SD)  ^‡^ Advice only presented to physicians of the intervention group in the directive study  PONV = postoperative nausea and vomiting; SD = standard deviation | | | | | | | |

# Table 3 – Crude risk-dependent PONV incidences between allocation groups of both impact studies

|  | | **Assistive Study** | | | **Directive Study** | | |
| --- | --- | --- | --- | --- | --- | --- | --- |
|  |  | | Care-as-Usual Group^†^ | Intervention Group^†^ |  | Care-as-Usual Group^†^ | Intervention Group^†^ |
| Predicted PONV risk in categories | N* | | (*n* = 4496) | (*n* = 3385) | N* | (*n* = 1022) | (*n* = 461) |
| <26% (0 antiemetics advised^‡^) | 521 | | 66 (23) | 42 (18) | 116 | 19 (23) | 10 (29) |
| 26-41% (1 antiemetic advised^‡^) | 2158 | | 443 (37) | 336 (35) | 432 | 123 (41) | 50 (38) |
| 41-62% (2 antiemetics advised^‡^) | 2515 | | 837 (59) | 626 (57) | 354 | 167 (64) | 48 (52) |
| >62% (3 antiemetics advised^‡^) | 471 | | 191 (71) | 145 (72) | 60 | 41 (82) | 5 (50) |
| * N represents the total number of non-missing observations for that characteristic  ^†^ Data represent absolute frequencies of PONV (%)  ^‡^ Advice only presented to physicians of the intervention group in the directive study  PONV = postoperative nausea and vomiting | | | | | | | |

# Table 4 – Regression analysis of the comparison of physicians’ administration of risk-dependent antiemetic prophylaxis between allocation groups of both the assistive and directive study.

| \|  \| **Complete case*** \| \| **Multiple imputation^‡^** \| \| --- \| --- \| --- \| --- \| \|  \| unadjusted \| confounder adjusted^†^ \| confounder adjusted^†^ \| \| Directive study \| 0.01 (-0.06 - 0.09) \| 0.04 (-0.06 - 0.14) \| 0.02 (-0.05 - 0.10) \| \| Intervention group \| 0.04 (-0.03 - 0.10) \| 0.03 (-0.05 - 0.10) \| 0.02 (-0.04 - 0.09) \| \| Recommendation categories^§^ \| **0.08 (0.06 - 0.10)** \| **-0.07 (-0.11 - -0.03)** \| **-0.06 (-0.09 - -0.02)** \| \| Interaction: directive study * intervention group \| 0.03 (-0.10 - 0.16) \| 0.05 (-0.11 - 0.20) \| 0.04 (-0.09 - 0.16) \| \| Interaction: directive study * categories ^§^ \| **0.11 (0.06 - 0.15)** \| **0.09 (0.03 - 0.14)** \| **0.10 (0.06 - 0.15)** \| \| Interaction: intervention group * categories ^§^ \| **0.15 (0.13 - 0.18)** \| **0.15 (0.12 - 0.19)** \| **0.15 (0.13 - 0.18)** \| \| Interaction: directive study * intervention group * categories ^§^ \| **0.31 (0.23 - 0.39)** \| **0.32 (0.23 - 0.41)** \| **0.32 (0.25 - 0.40)** \| \| Numbers represent regression coefficients with 95% confidence intervals. \| \| \| \| \| Bold numbers are statistically significant regression coefficients.  No unadjusted model is presented for multiple imputation as variables within the models were not missing. \| \| \| \| \| * Cases with missing variables were discarded \| \| \| \| \| ^†^ Adjusted for possible confounders: age (continuous, restricted cubic splines), gender, ASA class, current smoking, middle ear of abdominal surgery, history of PONV / motion sickness, use of inhalational anesthesia, procedure duration (continuous, restricted cubic splines) \| \| \| \| \| ^‡^ Using 10 imputation datasets \| \| \| \| \| ^§^ Regression coefficients represent an increase the number of administered prophylactic antiemetics per advised prophylactic antiemetics – i.e. per risk category \| \| \| \| |  |  |  |  |
| --- | --- | --- | --- | --- | --- | --- | --- | --- | --- | --- | --- | --- | --- | --- | --- | --- | --- | --- | --- | --- | --- | --- | --- | --- | --- | --- | --- | --- | --- | --- | --- | --- | --- | --- | --- | --- | --- | --- | --- | --- | --- | --- | --- | --- | --- | --- | --- | --- | --- | --- | --- | --- | --- | --- | --- | --- | --- | --- | --- | --- | --- | --- | --- | --- |

# Table 5 – Regression analysis of the comparison of the risk-dependent incidence of postoperative nausea and vomiting between allocation groups of both the assistive and directive study.

| \|  \| **Complete case*** \| \| **Multiple imputation^‡^** \| \| \| --- \| --- \| --- \| --- \| --- \| \|  \| unadjusted \| confounder adjusted^†^ \| unadjusted \| confounder adjusted^†^ \| \| Directive study \| **1.4 (1.1 - 1.7)** \| 1.3 (1.0 - 1.8) \| **1.3 (1.1 - 1.6)** \| **1.3 (1.1 - 1.6**) \| \| Intervention group \| 0.96 (0.83 - 1.1) \| 0.91 (0.75 - 1.1) \| 0.94 (0.83 - 1.1) \| 0.93 (0.81 - 1.1) \| \| Predicted risk^§^ \| **3.2 (2.8 - 3.7)** \| 0.47 (0.15 - 1.5) \| **2.7 (2.4 - 3.1)** \| 1.7 (1.0 – 3.0) \| \| Interaction: directive study * intervention group \| **0.55 (0.36 - 0.84)** \| 0.79 (0.59 - 1.1) \| **0.63 (0.45 - 0.88)** \| **0.63 (0.45 - 0.89)** \| \| Interaction: directive study * pred. risk^§^ \| 1.2 (0.84 - 1.7) \| **0.51 (0.32 - 0.84)** \| 0.96 (0.72 - 1.3) \| 0.99 (0.74 - 1.3) \| \| Interaction: intervention group * pred. risk^§^ \| 1.1 (0.85 - 1.3) \| 1.1 (0.71 - 1.7) \| 0.98 (0.82 - 1.2) \| 0.99 (0.83 - 1.2) \| \| Interaction: directive study * intervention group * pred. risk^§^ \| **0.42 (0.22 - 0.78)** \| 0.99 (0.77 - 1.3) \| **0.58 (0.36 - 0.92)** \| **0.54 (0.34 - 0.87)** \| \| Numbers represent odds ratios with 95% confidence intervals \| \| \| \| \| \| Bold numbers are statistically significant odds ratios \| \| \| \| \| \| * Cases with missing variables were discarded \| \| \| \| \| \| ^†^ Adjusted for possible confounders: age (continuous, restricted cubic splines, 5 knots), gender, ASA class, current smoking, middle ear of abdominal surgery, history of PONV / motion sickness, use of inhalational anesthesia, procedure duration (continuous, restricted cubic splines, 5 knots) \| \| \| \| \| \| ^‡^ Using 10 imputation datasets \| \| \| \| \| |  |  |  |  |
| --- | --- | --- | --- | --- | --- | --- | --- | --- | --- | --- | --- | --- | --- | --- | --- | --- | --- | --- | --- | --- | --- | --- | --- | --- | --- | --- | --- | --- | --- | --- | --- | --- | --- | --- | --- | --- | --- | --- | --- | --- | --- | --- | --- | --- | --- | --- | --- | --- | --- | --- | --- | --- | --- | --- | --- | --- | --- | --- | --- | --- | --- | --- | --- | --- | --- | --- | --- | --- | --- | --- | --- | --- | --- | --- |
| ^§^ Odds ratios represent predicted risks of 100% (a probability of 1) |  |  |  |  |

# References

1. Kappen TH, Moons KGM, van Wolfswinkel L, Kalkman CJ, Vergouwe Y, van Klei WA. Impact of risk assessments on prophylactic antiemetic prescription and the incidence of postoperative nausea and vomiting: a cluster-randomized trial. Anesthesiology. 2014;120:343–54. doi:10.1097/ALN.0000000000000009.

2. Kappen TH, Vergouwe Y, van Wolfswinkel L, Kalkman CJJ, Moons KGMGM, van Klei WAA. Impact of adding therapeutic recommendations to risk assessments from a prediction model for postoperative nausea and vomiting. Br J Anaesth. 2015;114:252–60. doi:10.1093/bja/aeu321.

3. Visser K, Hassink E a, Bonsel GJ, Moen J, Kalkman CJ. Randomized controlled trial of total intravenous anesthesia with propofol versus inhalation anesthesia with isoflurane-nitrous oxide: postoperative nausea with vomiting and economic analysis. Anesthesiology. 2001;95:616–26.

4. van den Bosch JE. Prediction of postoperative nausea and vomiting. [PhD Thesis]. 2006.

5. Kappen TH, Vergouwe Y, van Klei WA, van Wolfswinkel L, Kalkman CJ, Moons KGM. Adaptation of clinical prediction models for application in local settings. Med Decis Making. 2012;32:E1-10. doi:10.1177/0272989X12439755.

6. van den Bosch JE, Kalkman CJ, Vergouwe Y, Van Klei W a, Bonsel GJ, Grobbee DE, et al. Assessing the applicability of scoring systems for predicting postoperative nausea and vomiting. Anaesthesia. 2005;60:323–31. doi:10.1111/j.1365-2044.2005.04121.x.

7. Groenwold RHH, Klungel OH, Grobbee DE, Hoes AW. Selection of confounding variables should not be based on observed associations with exposure. Eur J Epidemiol. 2011;26:589–93. doi:10.1007/s10654-011-9606-1.

8. Harrell FE, Lee KL, Mark DB. Multivariable prognostic models: issues in developing models, evaluating assumptions and adequacy, and measuring and reducing errors. Stat Med. 1996;15:361–87. doi:10.1002/(SICI)1097-0258(19960229)15:4<361::AID-SIM168>3.0.CO;2-4.

9. Little RJA. Regression with missing x’s: A review. J Am Stat Assoc. 1992;87:1227–37. doi:10.2307/2290664.

10. van Buuren S, Boshuizen HC, Knook DL. Multiple imputation of missing blood pressure covariates in survival analysis. Stat Med. 1999;18:681–94.

11. Donders ART, van der Heijden GJMG, Stijnen T, Moons KGM. Review: a gentle introduction to imputation of missing values. J Clin Epidemiol. 2006;59:1087–91. doi:10.1016/j.jclinepi.2006.01.014.
